# Hepcidin secretion was not directly proportional to intracellular iron-loading in recombinant-TfR1 HepG2 cells: short communication

**DOI:** 10.1007/s11010-020-03716-8

**Published:** 2020-03-17

**Authors:** Kosha J. Mehta, Mark Busbridge, Vinood B. Patel, Sebastien Je. Farnaud

**Affiliations:** 1 Centre for Education, Faculty of Life Sciences and Medicine, King’s College London, London, UK; 2 School of Life Sciences, University of Westminster, London, UK; 3 Department of Clinical Biochemistry, Northwest London Pathology, Charing Cross Hospital, Imperial College Healthcare NHS Trust, London, UK; 4 Centre for Sport, Exercise and Life Sciences, Faculty of Health & Life Sciences, Coventry University, Coventry, UK

**Keywords:** Hepcidin, Iron, *HAMP*, Transferrin receptor, Iron-sensing, HepG2 cells

## Abstract

Hepcidin is the master regulator of systemic iron homeostasis and its dysregulation is observed in several chronic liver diseases. Unlike the extracellular iron-sensing mechanisms, the intracellular iron-sensing mechanisms in the hepatocytes that lead to hepcidin induction and secretion are incompletely understood. Here, we aimed to understand the direct role of intracellular iron-loading on hepcidin mRNA and peptide secretion using our previously characterised recombinant HepG2 cells that over-express the cell-surface iron-importer protein transferrin receptor-1. Gene expression of hepcidin (*HAMP*) was determined by real-time PCR. Intracellular iron levels and secreted hepcidin peptide levels were measured by ferrozine assay and immunoassay, respectively. These measurements were compared in the recombinant and wild-type HepG2 cells under basal conditions at 30 min, 2 h, 4 h and 24 h. Data showed that in the recombinant cells, intracellular iron content was higher than wild-type cells at 30 min (3.1-fold, *p* < 0.01), 2 h (4.6-fold, *p* < 0.01), 4 h (4.6-fold, *p* < 0.01) and 24 h (1.9-fold, *p* < 0.01). Hepcidin (*HAMP)* mRNA expression was higher than wild-type cells at 30 min (5.9-fold; *p* = 0.05) and 24 h (6.1-fold; *p* < 0.03), but at 4 h, the expression was lower than that in wild-type cells (*p* < 0.05). However, hepcidin secretion levels in the recombinant cells were similar to those in wild-type cells at all time-points, except at 4 h, when the level was lower than wild-type cells (*p* < 0.01). High intracellular iron in recombinant HepG2 cells did not proportionally increase hepcidin peptide secretion. This suggests a limited role of elevated intracellular iron in hepcidin secretion.

## Introduction

The hepatocyte-derived iron-hormone hepcidin is the main regulator of systemic iron homeostasis, whereby increased circulatory iron levels cause increased hepcidin secretion [1, 2]. Secreted hepcidin binds to the cell-surface iron-exporter protein ferroportin on various cell types and causes intracellular degradation of both hepcidin and ferroportin [3]. This impedes cellular iron efflux via ferroportin into the circulation and thereby prevents systemic iron elevation [4]. Lack of hepcidin or insufficient hepcidin production in response to systemic iron-loading characterises the genetic disorders of iron-overload hereditary hemochromatosis [5]. Moreover, hepcidin suppression together with ineffective erythropoiesis contributes to iron-loading anaemias like β-thalassemia [6], whereas excessive hepcidin induction is observed in iron-refractory iron deficiency anaemia [4] and in chronic inflammatory state such as in cancers [7]. Dysregulation of iron and hepcidin is also observed in other chronic liver conditions such as alcoholic liver disease, NAFLD-NASH, viral hepatitis and diabetes [8]. In chronic liver disease whether iron and hepcidin dysregulation is the cause, consequence or mediator of the pathology is not fully known, although hepcidin response to iron alterations appear to be functional [9]. Thus, fully understanding the effect of iron-loading on hepcidin secretion is of clinical value.

The extracellular iron-sensing mechanisms that induce hepcidin expression in the hepatocytes have been better characterised. Essentially, alterations in liver and plasma iron distinctly activate the BMP–SMAD pathway to induce hepcidin. Elevated liver iron is sensed by the hepatic endothelial cells that secrete paracrine signals and thereby simulate hepcidin induction in the hepatocytes, while plasma iron-sensing by the hepatocytes and subsequent hepcidin induction has been proposed to involve cell-surface proteins HFE and TFR2 [6, 10]. While most of such studies focussed on the extracellular iron-sensing by the hepatocytes, comparatively fewer studies have focussed on understanding the intracellular iron-sensing mechanisms that mediate hepcidin induction and secretion [11–14].

Along this line, the direct effect of high intracellular iron-loading on hepcidin mRNA expression and secretion need to be further investigated. This is important because previous studies suggested a role of stored intracellular iron in the induction of *HAMP* (gene encoding hepcidin) [15–17], and also proposed its role in the maturation of pro-hepcidin to bioactive hepcidin secreted in the circulation [18].

Thus, in this short study, we aimed to assess the exclusive effect of high intracellular iron on hepcidin under the hypothesis that the level of hepcidin secretion will be directly proportional to iron-loading. Accordingly, we examined *HAMP* mRNA and hepcidin peptide secretion in the recombinant transferrin receptor-1 (rec-TfR1) HepG2 cells, as previously created and characterised by our group [19]. These cells over-express the cell-surface iron-importer protein TfR1 that can allow high cellular loading.

Distinct from our previous study, here, we examined *HAMP* expression, hepcidin secretion, and intracellular iron levels in rec-TfR1 HepG2 cells under FCS-supplemented basal conditions over time in the absence of exogenous iron treatments, and in the absence of other cells types to exclude potential paracrine effects on hepcidin expression. Data were compared with some of our previously published data on Wild-type HepG2 cells [20] at each time-point.

## Materials and methods

### Cell culture and treatments

Wild-type (Wt) HepG2 cells (Health Protection Agency, UK) were maintained as previously described [11, 19, 20]. The maintenance medium used for both cell lines included foetal calf serum (FCS). To avoid the variability in the iron available to the cells due to differences in FCS batches, the same batch of FCS was used for all experiments. Rec-TfR1 HepG2 cells were previously created by our group and maintained accordingly (19). For treatments, Wt and rec-TfR1 HepG2 cells were seeded at a density of 5 × 10^5^ per well in 6-well plates in the maintenance medium; with and without hygromycin B for Wt and rec-TfR1 HepG2 cells, respectively) for 72 h (h). The antibiotic hygromycin B was required in the maintenance medium as a selection for exclusive growth of recombinant cells and not required for the growth and maintenance of Wt HepG2 cells. To ensure that before beginning experimental observations, the extracellular environment (medium) of both the cell lines was the same, Wt and rec-TfR1 HepG2 cells were incubated in hygromycin-free maintenance media. This removed media-imposed variability and eliminated the possibility of any hygromycin-posed interference in hepcidin peptide measurements by immunoassay, as hepcidin peptides had to be measured in the cell culture medium post the experimental time-point. Following 24 h of incubation in respective media, fresh hygromycin-free maintenance media were added to the wells. Cells were harvested for examination of parameters at 30 min, 2 h, 4 h and 24 h.

### Determination of intracellular iron content

Cellular iron content was determined by ferrozine assay [21]. This was normalised to protein content quantified by Bradford method and the iron levels were expressed as nmoles iron/mg protein, as previously described [11].

### Gene expression analysis

Primers (Invitrogen, UK) for gene expression analyses were ACAGCCAGACAGACGGCACGA (F) and TTCGCCTCTGGAACATGGGCATC (R) for *HAMP,* and GCCAAAAGGGTCATCATCTC (F) and GGTGCTAAGCAGTTGGTGGT (R) for *GAPDH* [19]. RNA was extracted, converted to cDNA using QuantiTect reverse transcription kit (Qiagen, UK) and assessed through real-time PCR by using Quantifast SYBR green kit (Qiagen, UK), as previously described [20]. Data were analysed by the relative quantification method, Delta-Delta *C*^t^ (∆∆*C*t) and expressed as $2^{{ - \Delta \Delta C_{{\text{t}}} }}$ [22].

### Hepcidin peptide measurement

The hepcidin peptide secreted in cell culture media was measured by a well-described immunoassay by Busbridge et al. [19, 23].

### Statistical analysis

Data analysis was performed using Student’s *t* test. The level of significance was set at *p* < 0.05. Data were presented as mean ± SEM.

## Results

### High intracellular iron in rec-TfR1 HepG2 cells

**Fig. 1:**
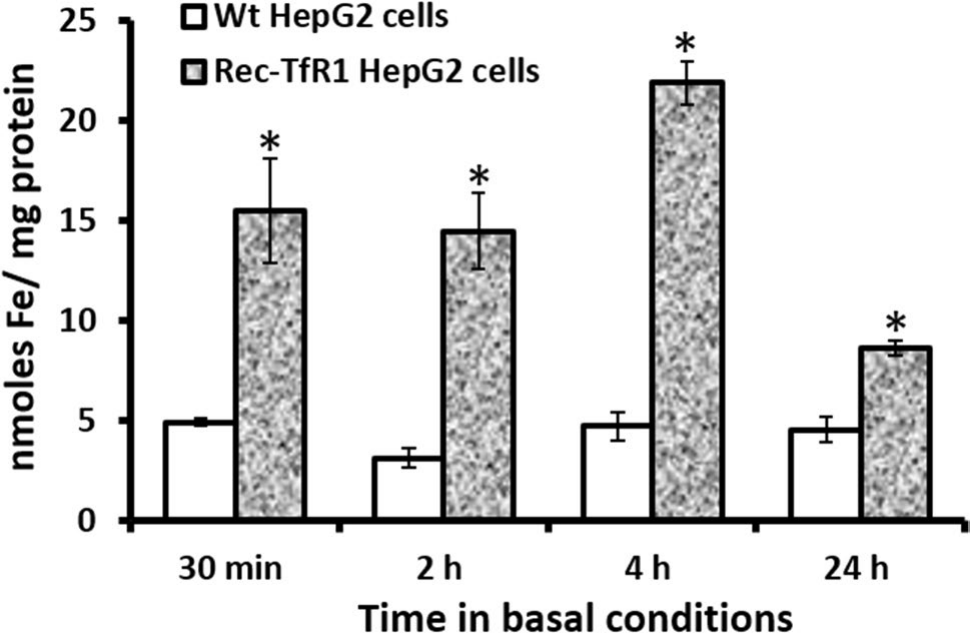
Cellular iron-loading in rec-TfR1 HepG2 cells. Intracellular iron levels in the recombinant cells were measured at various time-points in basal conditions by using the ferrozine assay and normalised to cellular protein content at corresponding time-points. *p < 0.01. Data are expressed as mean ± SEM (*n* = 3)

As expected, intracellular iron content in rec-TfR1 HepG2 cells was significantly higher than that in Wt-HepG2 cells at all time-points; 30 min (3.1-fold, *p* < 0.01), 2 h (4.6-fold, *p* < 0.01), 4 h (4.6-fold, *p* < 0.01) and 24 h (1.9-fold, *p* < 0.01) (Fig. 1).

### *HAMP* mRNA expression in rec-TfR1 HepG2 cells

**Fig. 2:**
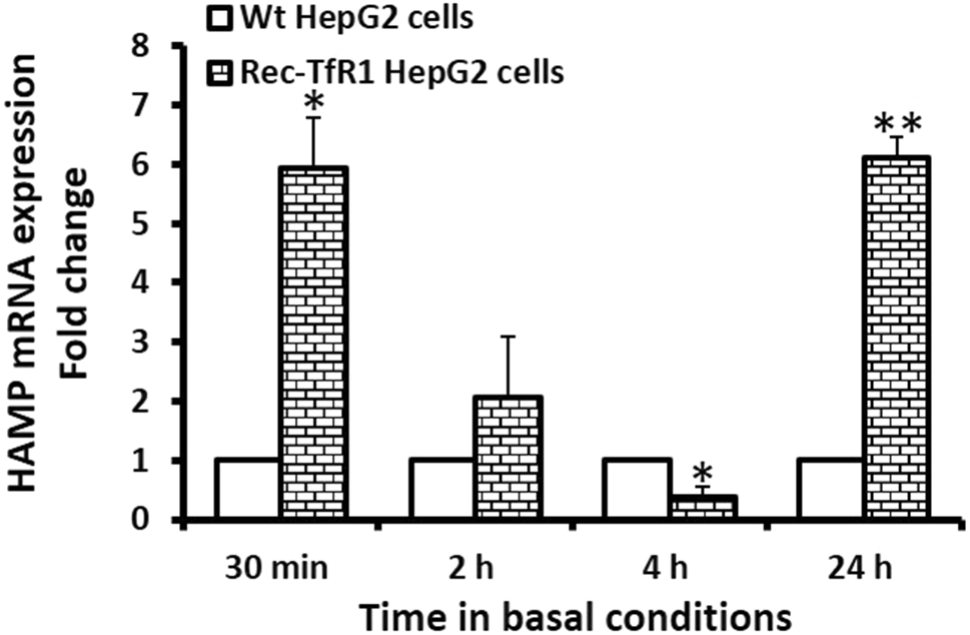
Hepcidin mRNA expression in rec-TfR1 HepG2 cells. *HAMP* mRNA expression was measured in the recombinant cells at various time-points in basal conditions by real-time PCR (normalised to *GAPDH*). *p ≤ 0.05, **p < 0.03. Data are expressed as mean ± SEM (*n* = 3)

In the rec-TfR1 HepG2 cells*, HAMP* mRNA expression showed an interesting pattern when compared to the Wt-HepG2 cells. In these recombinant cells, *HAMP* expression was significantly higher than that in Wt-HepG2 cells at 30 min (5.9-fold, *p* = 0.05) (Fig. 2). Following this, at 2 h, the expression did not significantly differ from Wt-HepG2 cells (Fig. 2), and at 4 h, it was lower than Wt-HepG2 cells (*p* < 0.03) (Fig. 2). At 24 h, *HAMP* expression restored its response to that observed at the 30 min time-point and was significantly higher than Wt-HepG2 cells (6.1-fold; *p* < 0.05) (Fig. 2).

### Hepcidin peptide secretion by rec-TfR1 HepG2 cells

**Fig. 3:**
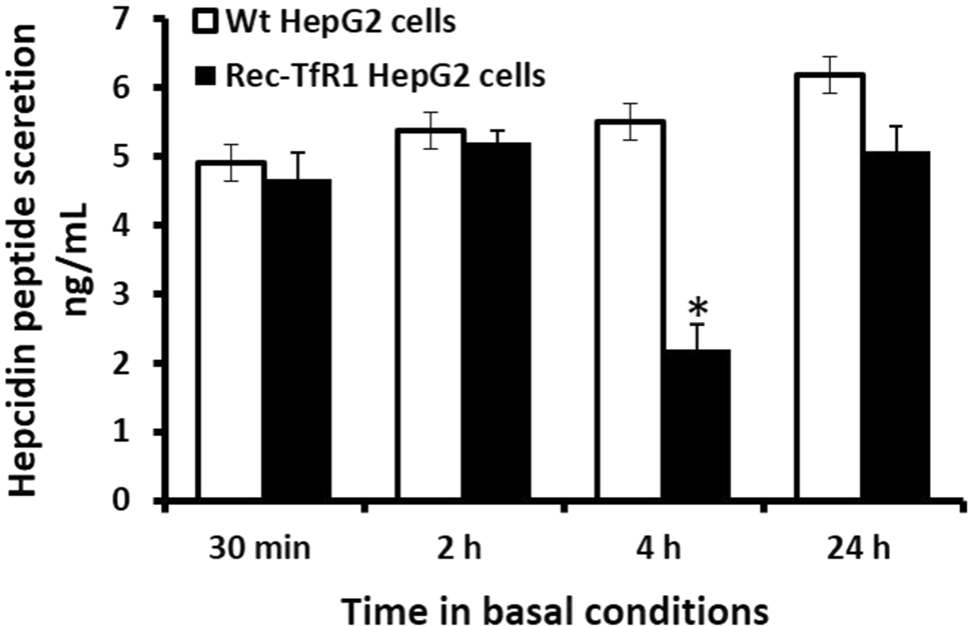
Hepcidin peptide secretion in rec-TfR1 HepG2 cells. Hepcidin peptides secreted into the culture medium were measured in the recombinant cells at various time-points in basal conditions, as mentioned in the methods section. *p < 0.01. Data are expressed as mean ± SEM (*n* = 3)

The pattern of hepcidin peptide secretion by the rec-TfR2 HepG2 cells was partly similar to the pattern of *HAMP* mRNA responses. These cells secreted hepcidin at similar levels to the Wt cells at 30 min and 2 h (Fig. 3). This was followed by comparatively lower levels of hepcidin secretion at 4 h (*p* < 0.01) (Fig. 3). Subsequently, at 24 h, hepcidin levels were restored (similar to Wt-HepG2 cells) and matched its previous response at 30 min (Fig. 3). Notably, secreted hepcidin peptide levels in the recombinant cells did not exceed levels in Wt-HepG2 cells at any time-point.

## Discussion

Under physiological conditions, increased hepatic hepcidin secretion in response to elevation in systemic iron levels is a well-established phenomenon [4]. Since hepcidin/iron dysregulation is observed in several hereditary and non-hereditary chronic liver conditions, and hepcidin is recognised as a useful biomarker for liver fibrosis and cirrhosis [8, 24], investigation on iron-induced hepcidin response is of immense clinical value.

Several studies have investigated on the role of iron on *HAMP* expression. Reviews by Kroot et al. [25], Wang et al. [26], Ganz [4] and Rishi et al. [27] elaborate the contribution of many groups in examining the significance of intracellular iron stores and its effect on *HAMP* induction via the BMP–SMAD pathway, and on overall transcriptional regulation of hepcidin. However, less is known about the post-translational life of hepcidin (i.e. mechanisms/modifications and regulation of secretion), except the role of furin convertase in the post-translational processing of 60-mer pro-hepcidin [28], autoregulation of *HAMP* by pro-hepcidin [29] and intracellular localisation of hepcidin in mycobacteria-containing phagosomes of infected interferon-gamma-activated cells that demonstrates the antibacterial nature of hepcidin [30]. Thus, there is scarcity of information on the regulation of 25-mer hepcidin at post-translational and secretion stages and the role of intracellular iron in these processes. Some reasons for comparatively fewer studies on this topic may include focus on other aspects of hepcidin in the early years following its discovery such as tackling the challenges in the detection and quantification of hepcidin in plasma and urine, difficulties in obtaining hepcidin antibodies due to its small size and limited availability of antigen [31], establishment of standardised protocols for hepcidin assays, identification and analysis of hepcidin in species apart from mammals/human [32], examination of hepcidin-inducing signalling pathways [27], clinical characterisation of differences in hepcidin levels in the different types of hereditary hemochromatosis [33], analysis of hepcidin levels and hepcidin:ferritin ratios for disease diagnosis [8, 25, 34] and usage of synthetic hepcidin in therapeutics [35].

While the mediators of extracellular iron-sensing by the hepatocytes have been well studied [6, 36, 37], intracellular iron-sensing mechanisms in the hepatocytes that affect the induction and secretion of hepcidin are not fully understood. Whether excess intracellular iron per se plays a direct role in hepcidin induction and secretion remains enigmatic.

Therefore, in this short study, we examined the exclusive effect of high intracellular iron on *HAMP* mRNA expression and hepcidin peptide secretion in recombinant HepG2 cells under basal conditions, in the absence of exogenous iron-loading and paracrine effects exerted by other liver cell types. To achieve elevation in cellular iron, we used our previously characterised rec-TfR1 HepG2 cells, which over-express cell-surface TfR1 [19].

We confirmed cellular iron-loading in the rec-TfR1 HepG2 cells to ensure that the observed effects on hepcidin were specifically due to elevated intracellular iron (Fig. 1). Previously, it was proposed that iron may participate in the maturation of pro-hepcidin into secreted bioactive hepcidin [18], indirectly implying a direct relationship between intracellular iron and hepcidin secretion levels at the post-translational level. Accordingly, here, we hypothesised that hepcidin secretion will be directly proportional to cellular iron-loading. However, unlike *HAMP* mRNA expression (Fig. 2), hepcidin peptide secretion in the recombinant cells did not exceed levels in Wt cells at any time-point (Fig. 3), despite their several-fold higher intracellular iron content (Fig. 1) and higher *HAMP* mRNA expression at 30 min and 24 h (Fig. 2). Thus, hepcidin secretion was not directly proportional to intracellular iron-loading (Figs.1, 3), despite the absence of mutations in iron-related genes, in which case the disproportionality would be expected. These data are in line with our previous observation in these cells where holo-transferrin treatments (extracellular iron) combined with increased intracellular iron content did not allow hepcidin secretion in these cells to surpass the levels in Wt cells [19].

Although hepcidin secretion and intracellular iron did not show direct proportionality as expected, comparison of this data in rec-TfR1 HepG2 with our previously obtained data in CHO-TRVb1 cells [20] may partially support the proposed role of iron in hepcidin secretion [18]. The CHO TRVb1 cells are well characterised Chinese hamster ovary transferrin receptor variant cells that lack endogenous TfR1 and express iron-response-element-depleted human TFRC mRNA [38], and over-express human TFR1 on cell surface [20]. Previously, the CHO-TRVb1 cells did not differ from the Wt-HepG2 cells in cellular iron content but showed lower hepcidin secretion than Wt-HepG2 cells at the same time-points as in this study [20]. Here, the rec-TfR1 HepG2 cells showed substantially higher intracellular iron than Wt-HepG2 cells (Fig. 1), and although hepcidin secretion did not exceed levels in Wt cells (Fig. 3), the levels were higher than that in CHO-TRVb1 cells [20]. Thus, the comparatively higher level of hepcidin secretion from the rec-TfR1 HepG2 than CHO-TRVb1 cells, in the perspective of corresponding comparisons with Wt-HepG2 cells, may be partly attributed to the higher cellular iron-loading in the former cells, assuming the genetic differences between the cell lines did not influence hepcidin response to iron. A limited but positive role of intracellular iron in hepcidin secretion can be envisaged.

Data showed higher *HAMP* mRNA expression in the rec-TfR1 HepG2 cells compared to Wt cells at 30 min and 24 h (Fig. 2). This could be attributed to the higher cellular iron content in these cells (Fig. 1); the mechanism likely mediated via the previously established endogenous BMP6-related iron-sensing mechanism [12, 13]. The decrement in *HAMP* mRNA expression at 4 h (Fig. 2), which reflected in reduced hepcidin peptide secretion at 4 h (Fig. 3) followed by the simultaneous restoration of levels at 24 h (Figs. 2, 3) is interesting and suggests a specific time-based pattern of *HAMP* regulation in these recombinant cells. As observed here, several other studies have shown time-dependent alterations/regulation of hepcidin. For example, Lakhal-Littleton et al. observed that in control mice cardiomyocytes, *HAMP* mRNA expression gradually elevated and peaked at 8 h, then decreased at 16 h when the levels were similar to that at 4 h and remained unaltered thereafter up to 24 h. Similarly, hepcidin peptide levels generally increased and peaked at 4 h, then dropped and subtly increased again, restoring levels to that at 4 h [39]. Serum hepcidin levels in healthy individuals have also shown time-related alterations and demonstrated diurnal rhythm. For instance, Ganz et al. reported that in comparison to the morning (8 am), hepcidin was higher at noon and evening [40]. Girelli et al. showed that hepcidin gradually increased and peaked at 12 h and returned to baseline levels at 24 h [41]. Troutt et al. reinforced that hepcidin demonstrated a diurnal rhythm with lowest concentrations in the morning, followed by gradual increase during the day, and then a decline in late evening [42]. Schaap et al. further studied this and suggested that ferritin was the determinant of basal hepcidin levels, and diurnal rhythm rather than dietary iron mediated hepcidin alterations [43].

While these examples of time-related hepcidin regulation spanned hours, hepcidin response has been observed to cover several days. For instance, in rats, there was a delay of about 4 days between stimulated erythropoiesis and the elevation in intestinal iron absorption. It was concluded that this was due to the lag in hepcidin response and thereby the delay in its action on mature villus enterocytes [44]. Other examples include malaria infection in mice, in which liver hepcidin expression increased during early stages to offer protection against infection and then decreased during late stages [45]. In human volunteers, hepcidin levels elevated after 3 days of fasting and refeeding reversed this effect. It was postulated that during fasting, erythropoiesis was suppressed to withhold tissue iron for important cellular processes such as oxidative respiration (iron-dependent cytochrome system in the mitochondria). This decrement in erythropoiesis could be sensed by the liver resulting in elevated hepcidin synthesis and secretion [42]. Also, during menstrual cycle, there is an initial decrease in hepcidin during menstruation, followed by elevation and stabilisation in the second half of the cycle [46, 47]**.** These scenarios reflect the factors on which hepcidin synthesis and secretion depend (such as iron status, inflammation, fasting, diurnal rhythm) and these form the physiological basis for time-dependent regulation of hepcidin. Thus, such time-dependent alteration/regulation of hepcidin exists to allow hepcidin responses to be executed at specific times and alter the responses according to body conditions and circadian rhythm.

The reduction in *HAMP* mRNA expression in the rec-TfR1 HepG2 cells (Fig. 2) could be partly executed by the over-expression of TfR1 in these cells. This is possible because a similar response of reduced hepcidin secretion was observed in the CHO-TRVb1 cells that also over-express human TfR1 [20]. Thus, the existence of a TfR1-related mechanism of hepcidin regulation can be envisioned. The proposed role of TfR1 is further strengthened by the fact that under physiological conditions, iron deficiency reduces hepcidin levels [48], but here, the recombinant cells were not iron deficient (Fig. 1) and still a reduced hepcidin response was observed at 4 h (Figs. 2, 3). A contradictory relationship between iron and hepcidin was observed in the rec-TfR1 HepG2 cells whereby at 4 h, when the intracellular iron content was at its highest (Fig. 1), *HAMP* mRNA and secretion levels were at their lowest (Figs. 2, 3). This suggests that the BMP-6-mediated intracellular iron-sensing mechanism to elevate hepcidin expression may be overruled by other regulatory mechanisms (partly by the putative regulatory effect of TfR1) at this time-point. However, these putative mechanisms were surpassed at a longer time-point of 24 h when intracellular iron content was at its lowest (Fig. 1), but *HAMP* mRNA response was elevated (Fig. 2) and hepcidin secretion was restored (Fig. 3). Thus, an endogenous time-based regulatory effect of TfR1 on hepcidin expression can be envisaged and needs to be investigated further. Interestingly, these cells resemble the scenario created due to the mutation in *TFRC* (gene encoding TfR1) in human, as recently identified, which leads to increased cell-surface TfR1 [49]. Our data may partly support the recently published data indicating the essential role of TfR1 in tuning hepcidin expression based on the level of iron-loading in hepatocytes and reiterates the inhibitory function of TfR1 on hepcidin expression [50].

One physiological reason for the disconnect between hepcidin mRNA and protein levels could be to generate an intracellular stored stock of the peptide so that it is ready to be released in the circulation immediately upon stimulus and/or allow a time-related release, as observed in several clinical studies [40–43]. To our knowledge, such a mechanism involving hepcidin storage/accumulation in intracellular vesicles and its regulated release has not been demonstrated so far but does not exclude this possibility either; particularly when hepcidin has been described as an iron-hormone [51]. While it remains to be demonstrated, this putative mechanism may be similar to that in insulin, where insulin is stored intracellularly in vesicles with zinc in the form of dense clustered granules and released upon stimulation by circulating nutrients. Insulin exocytosis is stimulated by elevation in intracellular calcium levels [52]. Similarly, it is possible that hepcidin secretion may be enhanced due to elevation in intracellular iron. This hypothesis formed the basis for the work presented here.

Another reason for the disconnect or gap between hepcidin mRNA and protein levels could be to accommodate for any regulation at post-translational or secretion stage to mediate fine tuning of hepcidin exocytosis. Again, this is a postulation and remains to be proven. Note that systemic hepcidin levels are not only determined by systemic and hepatocellular iron but also by hypoxia [53], erythropoiesis [54], inflammation (IL-6, TGF-β1) [55], diurnal/circadian rhythm [42], growth hormone [42], tissue specificity, gluconeogenic signals [56] and pathological status [8]. Thus, it is possible that the disconnect between *HAMP* mRNA and peptide levels (particularly in the hepatocytes) exist to accommodate the alterations in cellular and physiological conditions, and thereby contribute to the fine tuning of hepcidin in response to the aforementioned factors.

With regards to the possible effect of cell culture conditions on hepcidin production, here, the culture conditions deployed (including the maintenance medium) were the most basic, commonly used and well established for the growth and maintenance of HepG2 cells. Thus, it is unlikely that cell culture condition may be a factor in altering metabolic pathways or epigenetic regulation related to hepcidin. Moreover, the culture conditions for both, the Wt (control) and recombinant HepG2 cells (test) were the same before beginning experimental observations, which may have nullified any differences between the control and test culture conditions. However, paracrine/autocrine signalling, whereby these cells may release signalling molecules and target itself or neighbouring cells may affect hepcidin production. For example, inflammatory cytokine such as interleukin (IL)-6, that may be released by these cells could increase *HAMP* transcription (hepcidin is a type II acute phase protein) [57]. In contrast, cytokines involved in the type I inflammatory response such as tumour necrosis factor-α inhibit hepcidin expression [58], while IL1α does not induce hepcidin [59]. These inflammatory cytokines were not measured in this study. Hence their exact paracrine effect on hepcidin could not be determined. Similarly, growth factors like hepatocyte growth factor and epidermal growth factor can inhibit the iron- and BMP6-mediated *HAMP* induction [59]. Sex hormone estrogen reduces circulating hepcidin [60] but treatment of HepG2 cells with E2 has shown to increase *HAMP* mRNA expression [61], while testosterone has shown to decrease *HAMP* transcription [62]. However, these are relevant under physiological conditions and not under the in vitro experimental conditions deployed here.

Our objective was to examine whether hepcidin secretion was directly proportional to the elevation in intracellular iron. So far, we have not come across a study that examines the exclusive effect of excess intracellular iron on hepcidin secretion levels, which makes the concept of this study novel and interesting. Indeed, hepcidin peptide levels under physiological and pathological conditions, and in response to iron dosage have been measured in numerous clinical studies, in animal models and in in vitro studies [8, 37, 40, 41, 57, 63, 64]. Also, several studies have contributed to our knowledge on hepcidin regulation at transcriptional level [59]. However, the aim of such elite studies wasn’t to understand hepcidin secretion mechanisms and determine the role of iron in secretion or in the maturation of pro-hepcidin to bioactive hepcidin. Thus, studies need to be conducted on understanding the mechanism and regulation of hepcidin secretion.

Future work could test the hypothesis that TFR1 contributes to hepcidin regulation. Hepcidin mRNA, intracellular hepcidin peptide and levels of secreted hepcidin could be determined overtime under iron-loaded, basal and iron-depleted conditions. This could be done in Wt HepG2 cells/primary hepatocytes and compared with corresponding data from cell lines overexpressing human TFR1 such as the rec-TfR1 HepG2 cells. Comparison of intracellular hepcidin levels between the two cell lines under basal, iron-loaded and iron-depleted conditions and examining whether intracellular hepcidin peptides inhibit *HAMP* transcription may provide further insights into hepcidin mRNA regulation by hepcidin peptide.

## Conclusion

Data showed that high intracellular iron levels in recombinant HepG2 cells elevated *HAMP* mRNA expression but did not proportionally increase hepcidin peptide secretion. This suggests a limited role of iron in hepcidin peptide secretion. A putative role of TfR1 in hepcidin regulation can be envisioned.
